# Basal Transcription Factor 3 Plays an Important Role in Seed Germination and Seedling Growth of Rice

**DOI:** 10.1155/2014/465739

**Published:** 2014-05-29

**Authors:** Wenyi Wang, Mengyun Xu, Ya Wang, Muhammad Jamil

**Affiliations:** ^1^Institute of Crop Science, College of Agriculture and Biotechnology, Zhejiang University, Yu-Hang-Tang Road 388, Hangzhou 310058, China; ^2^Department of Biotechnology and Genetic Engineering, Kohat University of Science and Technology, Kohat 26000, Pakistan

## Abstract

BTF3 has been recognized to be involved in plant growth and development. But its function remains mostly unknown during seed germination and seedling stage. Here, we have analyzed OsBTF3-related sequences in *Oryza sativa* L. subspecies, japonica, which resembles with the conserved domain of a nascent polypeptide associated complex (NAC) with different homologs of OsBTF3 and human BTF3. Inhibition of *Osj10gBTF3* has led to considerable morphological changes during seed germination and seedling growth. Germination percentage was not influenced by the application of GA_3_, ABA, and NaCl but all concentrations caused wild-type (WT) seeds to germinate more rapidly than the RNAi (*Osj10gBTF3* ^Ri^) transgenic lines. Seedling inhibition was more severe in the *Osj10gBTF3* ^Ri^ seedlings compared with their WT especially when treated with 100 or 200 *μ*M GA_3_; 50% reduction in shoots was observed in *Osj10gBTF3* ^Ri^ seedlings. The expression of *Osj3g1BTF3*, *Osj3g2BTF3* and *Osj10gBTF3* was primarily constitutive and generally modulated by NaCl, ABA, and GA_3_ stresses in both *Osj10gBTF3* ^Ri^ lines and WT at the early seedling stage, suggesting that *Osj3g1BTF3* and *Osj10gBTF3* are much similar but different from *Osj3g2BTF3* in biological function. These results show that OsBTF3 plays an important role in seed germination and seedling growth gives a new perception demonstrating that more multifaceted regulatory functions are linked with BTF3 in plants.

## 1. Introduction

Different transcription factors (TFs) play an important role in multiple physiological mechanisms such as cell cycle progression, metabolism, growth, development, and reproduction [1, 2]. Among these TFs, the basal transcription factors 3 (BTF3) is one of the extremely important transcription factors due to their role in various biotic and abiotic stress processes [3–6] and different physiological and developmental mechanisms such as ionic homeostasis [7], photosynthetic rate [8], and pollen development [6] in plants.

BTF3 was basically found in HeLa cell that is essential for RNA polymerase II dependent transcription [9]. The BTF3 codes two isoforms, BTF3a and BTF3b, in the human, as an outcome of alternative splicing. BTF3a encodes for a protein with complete characteristics of BTF3 and stimulates transcription, while BTF3b encodes a shortened form of BTF3a, as it is missing the first 44 amino-terminal extension. Afterward, BTF3b was documented as a component of the nascent polypeptide associated complex (NAC). It consists of two subunits, that is, *α*NAC and *β*NAC [10]. BTF3 is a *β*-subunit of the NAC that has been participating in regulating protein localization during translation [11].

BTF3 in plant lacks the N-terminals which exist in the human BTF3a form. But, the N-terminals extension of the plant proteins interacts with that of the transcriptionally dormant BTF3b isoform [12]. In* Arabidopsis*, AtBTF3 coincides with the translation initiation factor (iso) 4E (eIFiso4E) and formed the translation initiation complex eIF(iso)4F [12].* NbBTF3* silenced plants show an abnormally developed phenotype in* Nicotiana benthamiana*. Similarly, in* TaBTF3*-silenced wheat transgenic plants, Ma et al. [13] demonstrated that the transcripts of the mitochondrial and chloroplast encoded genes were noticeably decreased. In* Capsicum annuum*, BTF3 gene was observed to play a role in hypersensitive response (HR) cell death and might function as TFs in the nucleus via transcriptional regulation of HR-related gene expression [14].

Recently, in rice, Wang et al. [6] have demonstrated that BTF3 plays important role in plant growth and development. However, the function of different variants of OsBTF3 in seed germination and seedling growth is not yet documented. In the present work, a cDNA sequence of a BTF3-like gene (*Osj10gBTF3*) was extracted, and its function examined via transgenic strategies to recognize the role it played in seed germination and seedling growth in rice. We also attempt to investigate that how the OsBTF3s were transcriptionally regulated by salt, GA_3_, and ABA stresses during seed germination and seedling stage in* Osj10gBTF3* ^Ri^ lines and wild type.

## 2. Materials and Methods

### 2.1. Isolation of* Osj10gBTF3* and Generation RNAi Repression Lines

RNAs extractions, the full length of cDNA synthesis, were carried out using the BTF3 gene-specific primers via procedures as described by Wang et al. [6]. The amplified products of cDNA were cloned into a pMD18-T vector (TaKaRa). RNAi repression vector was constructed as suggested by Wang et al. [6]. The resultant vector was incorporated into the EHA105 (*A. tumefaciens* strain), which was used to infect rice embryogenic calli raised from mature seeds of Nipponbare to produce transformed calli. Transformants were screened using primers specific for the hygromycin B phosphor transferase gene (*Hpt*) by PCR amplification as demonstrated by Wang et al. [6].

### 2.2. Seed Germination and Seedling Growth

Seeds of T_1_ *Osj10gBTF3* ^Ri^ and wild-type (WT) of* Oryza sativa* L. subspecies* japonica* line Nipponbare were germinated on double filter paper in Petri plates treated with 0 (distilled water), 50 and 100 mM NaCl, 100 and 200 *μ*M GA_3_, and 5 and 10 *μ*M ABA. The seeds were incubated at 30 ± 2°C in dark condition in growth chamber. Seed germination was counted at an interval of 12 hours up to 7 days, up to emergence of ~2 mm radical and germination percentage was calculated when there was 50% of seed germination. Seedlings after 15 days were taken and separated into shoots and roots for measuring root and shoot lengths.

### 2.3. RNA Isolation from Different Tissues

RNA was extracted from the fresh roots and shoots of* Osj10gBTF3* ^Ri^ lines and WT under normal and stress conditions with Trizol reagent (Invitrogen) by following the manufacturer's instructions. Full length of cDNA synthesis of first-strand was done with the M-MLV first-strand synthesis system.

### 2.4. Expression Analysis Using Real-Time PCR

To check the expression of* Osj3g1BTF3*,* Osj3g2BTF3,* and* Osj10gBTF3*, real-time quantitative PCR (q-PCR) was used. The target gene names and their primers for the q-PCR are listed in Supplementary Table 2 (see Supplementary Material available online at http://dx.doi.org/10.1155/2014/465739). Q-PCR was done using the method of Wang et al. [6]. The relative expression levels of* Osj3g1BTF3*,* Osj3g2BTF3,* and* Osj10gBTF3* in root and shoot were calculated using the 2^−ΔΔ *C*_*T*_^ method [15]. Here, ΔΔ*C*_*T*_ = (*C*_*T*__(Target,test)_ − *C*_*T*__(ubiquitine,test)_)−(*C*_*T*__(Target,calibrator)_ − *C*_*T*__(ubiquitine,calibrator)_). The *C*_*T*_ (cycle threshold) value was the average value of the three different independent samples for both target and reference genes.

### 2.5. Statistical Analysis

The data were analyzed by one-way analysis of variance (ANOVA), and mean values were separated by least significant difference (LSD) at the 5% and 1% probability level using Statistical software (Sigmaplot 10.0.). Sequences of different variants of OsBTF3 were aligned by using ClustalX, while MEGA 5.0 was used for construction of phylogenetic tree.

## 3. Results

### 3.1. OsBTF3 Sequence and Phylogenetic Tree

**Figure 1:**
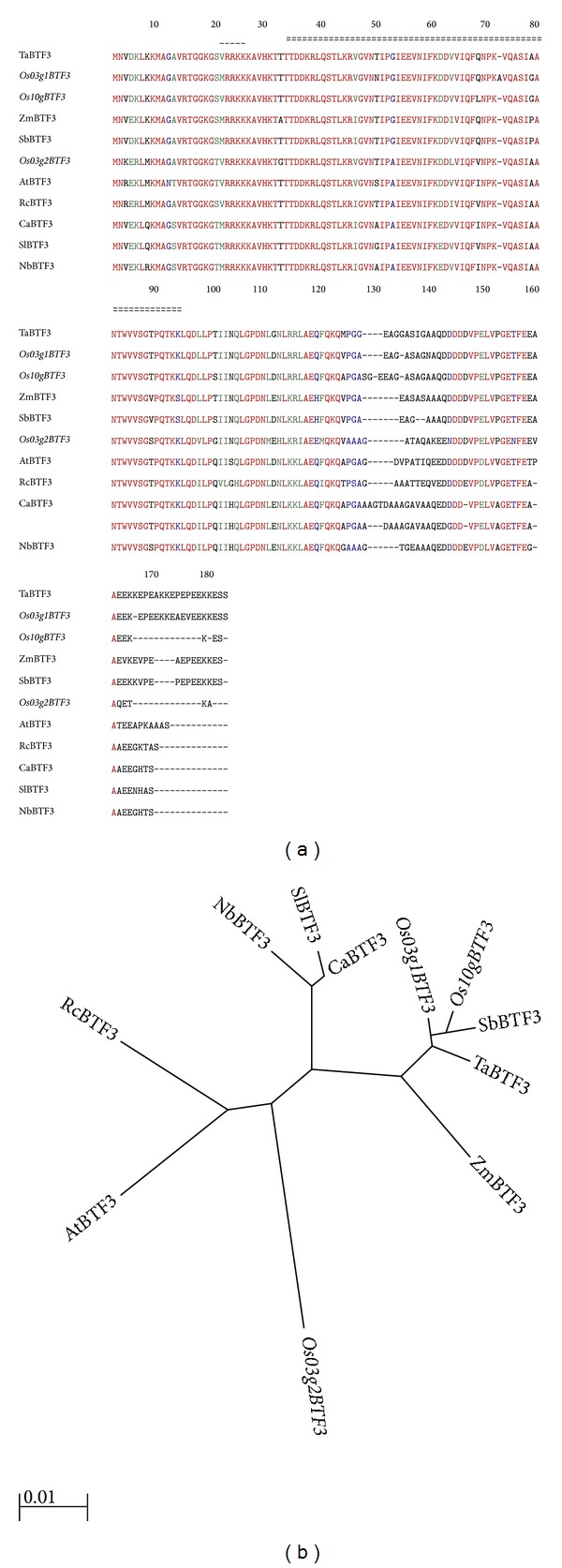
Amino acid sequence comparison (a) and phylogenetic tree (b) of different variants of OsBTF3. A deduced amino acid sequence of different variants of OsBTF3 and alignment with related sequences of different plants. The residues conserved among the compared sequences are in red colour. The overline (---) indicates a putative nuclear localization signal sequence, while the double dashed line (==) indicates the NAC domain. Gene Bank accession numbers for the proteins in the alignment are as follows:* Arabidopsis thaliana* BTF3 (AEE29647),* Nicotiana benthamiana* BTF3 (ABE01085),* Oryza sativa 10gBTF3* (NP_001064883),* Oryza sativa 3g1BTF3* (NP_001048709),* Oryza sativa 3g2BTF3* (NP_001051911),* Capsicum annuum* BTF3 (ABM55742),* Ricinus communis* BTF3 (EEF34688),* Solanum lycopersicum* BTF3 (NP_001234229),* Triticum aestivum* BTF3 (AFV31408),* Zea mays* BTF3 (ACG28870), and* Sorghum bicolor* BTF3 (EER93008). The tree was constructed using MEGA5 software.

The protein sequences of different variants of OsBTF3 were aligned with the related sequences from* Arabidopsis* (AtBTF3, AEE29647), wheat (TaBTF3, AFV31408), sorghum (SbBTF3, EER93008), maize (ZmBTF3, ACG28870),* Ricinus communis* (RcBTF3, EEF34688),* Capsicumvannum* (CaBTF3, ABM55742),* Solanum lycopersicum* (SlBTF3, NP_001234229), and* Nicotiana benthamiana* (NbBTF3, ABE01085) (Supplementary Table 1). Protein sequence analysis showed that different variants of OsBTF3 such as* Osj10gBTF3* and* Osj3g1BTF3* contained a putative mitochondrial matrix targeting sequence (MMTS) (residues 1–15, MNVDKLKKMAGAVRT), a putative nuclear localization signal (NLS) (residues 22–25, RRKK), a conserved NAC domain (residues 33–97), and a putative ER-retention/retrieval signal (ERRS) (residues 161–164, AEEK), while* Osj3g2BTF3* lack MMTS or ERRS (Figure 1(a)). It means that these two genes having different genomic loci are either cytosolic or a nucleus proteins. These results show that* Osj10gBTF3* and* Osj3g1BTF3* are probably differing from* Osj3g2BTF3* in subcellular localization and other biological function.

Phylogenetic analysis (Figure 1(b)) was performed between different variants of OsBTF3 and BTF3-like members from other plant species. The analysis showed that* Osj10gBTF3* is more strongly associated to* Osj3g1BTF3*, followed by other BTF3 homologs of the plant species. The BTF3 homologs of monocot and dicot species form different clades. Additionally,* Osj3g2BTF3* has a closer association to the homologs of dicot species AtBTF3 and RcBTF3 than* Osj3g1BTF3* and* Osj10gBTF3.* These results indicate that there may be functional variation between* Osj3g1BTF3*,* Osj10gBTF3,* and* Osj3g2BTF3* of BTF3 proteins.

### 3.2. Inhibition of* Osj10gBTF3* Causes Phenotypic Changes

**Figure 2:**
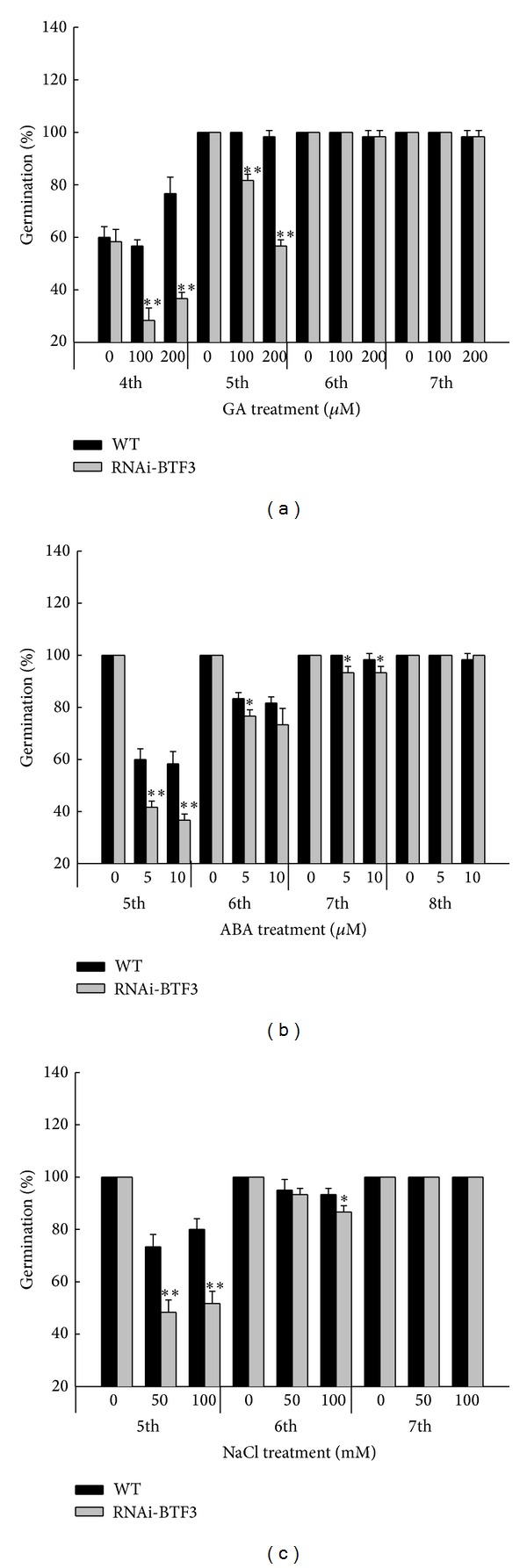
Germination of* Osj10gBTF3* ^Ri^ lines and wild-type (WT) rice seeds treated with different concentrations of GA_3_, ABA, and NaCl. Germination readings were taken after 4th, 5th, 6th, 7th, and 8th days. Error bars indicate the S.D. (*n* = 30). *P ≤ 0.05, **P ≤ 0.01 (Student's *t*-test).

The functional analysis of BTF3 was investigated using repression and overexpression strategies. Different independent* Osj10gBTF3* overexpressed lines were produced before the application of the RNAi construct for functional analysis of* Osj10gBTF3* using the overexpression constructs. But most of these lines did not show any significant change in phenotype; therefore, we mainly focused on RNAi inhibition analysis.* Osj10gBTF3* ^Ri^ transgenic seeds were investigated for their capability to germinate, mainly in the presence of different concentrations of GA_3_, ABA, and NaCl. Though the final germination percentage was not affected by the application of GA_3_, ABA, and NaCl but all the levels of GA_3_, ABA, and NaCl caused WT seeds to germinate more rapidly than the* Osj10gBTF3* ^Ri^ transgenic lines and their mean germination rate reduced with increased GA_3_, ABA, and NaCl concentrations (Figure 2). It is worth mentioning here that the decrease in germination rate was more severe in the GA_3_ treated seeds as compared to ABA and NaCl. These results demonstrate that* Osj10gBTF3* ^Ri^ transgenic lines are more sensitive to GA_3_ than ABA and NaCl.

**Figure 3:**
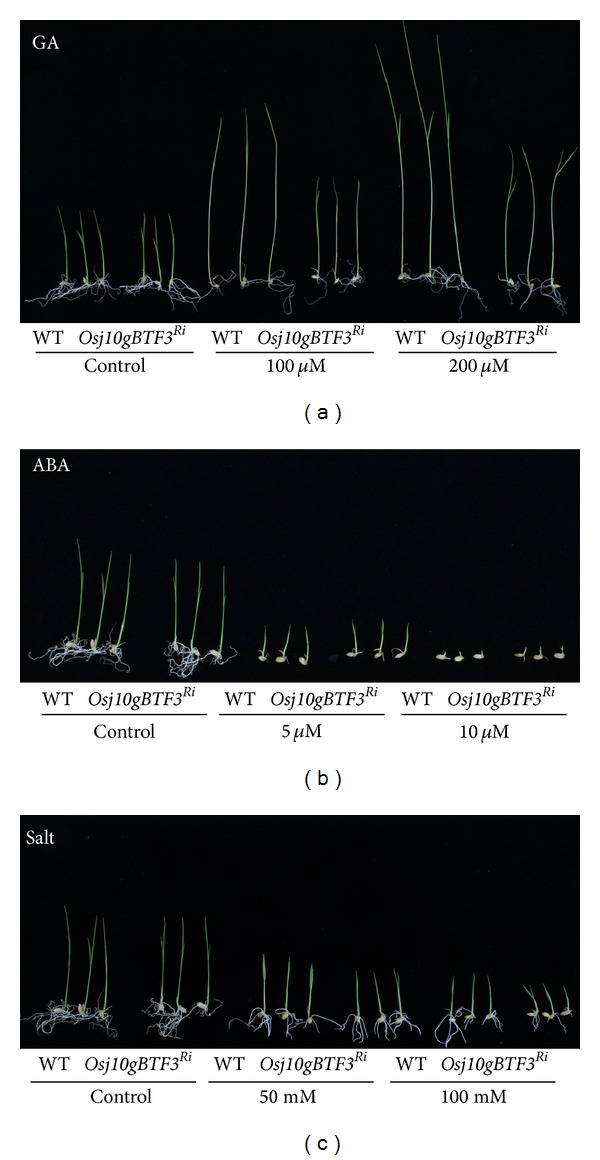
Comparison of* Osj10gBTF3* ^Ri^ lines and wild-type (WT) rice seeds grown under various concentrations of GA_3_, ABA, and NaCl.

**Figure 4:**
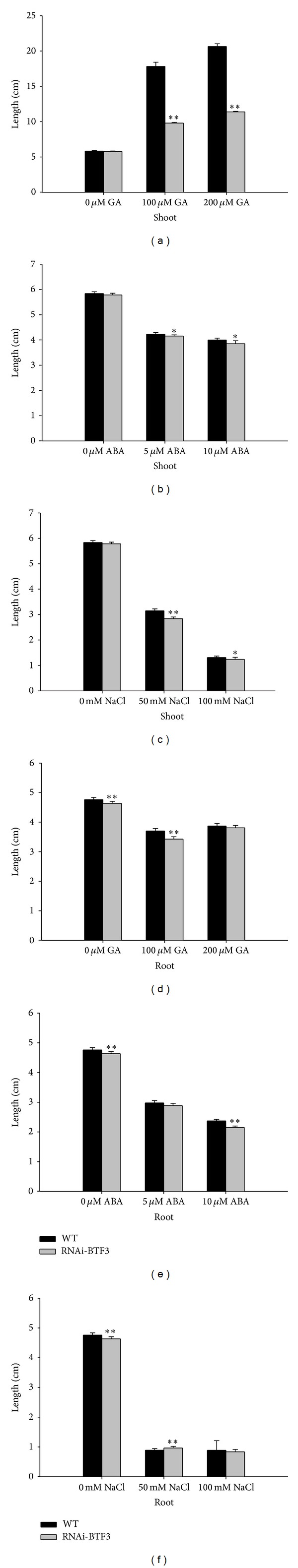
Response of* Osj10gBTF3* ^Ri^ lines and wild-type (WT) seedlings to different concentrations of GA_3_, ABA, and NaCl after 15 d of germination. Error bars indicate the S.D. (*n* = 30). *P ≤ 0.05, **P ≤ 0.01 (Student's *t*-test).

The seedling growth of WT and* Osj10gBTF3* ^Ri^ lines showed a different trend of variations in response to GA_3_, ABA, and salt treatments (Figure 3). The root and shoot lengths decreased in response to increasing concentrations of ABA and NaCl (Figure 4). However, NaCl had a more negative effect on root and shoot than ABA. On the other hand, GA_3_ did not induce significant changes in root with increasing concentration of GA_3_; however, a considerable increase in the shoot was observed with the increasing level of GA_3_. It was worth observing that the repression was more serious in the* Osj10gBTF3* ^Ri^ seedlings compared with their WT. When treated with 100 or 200 *μ*M GA_3_, 50% reduction in shoots was observed in* Osj10gBTF3* ^Ri^ seedlings compared to their WT (Figure 4). However, shoot length was increased in* Osj10gBTF3* ^Ri^ seedlings when treated with 50 mM NaCl. These results demonstrate that inhibition of BTF3 caused significant phenotypic changes during seed germination and seedling growth; however, these changes were more serious when treated with GA_3_ as compared to ABA and NaCl.

To assess whether these phenotypic changes in* Osj10gBTF3* ^Ri^ seedling were caused by RNAi inhibition, real-time quantitative PCR analyses were carried out using the OsBTF3-specific primers (Supplementary Table 2) to check the regulation of different variants of OsBTF3 in root and shoot at seedling stage under ABA (5 and 10 *μ*M), GA_3_ (100 and 200 *μ*M), and (50 and 100 mM) salt stresses. The ubiquitine mRNA level was used as a positive control.

**Figure 5:**
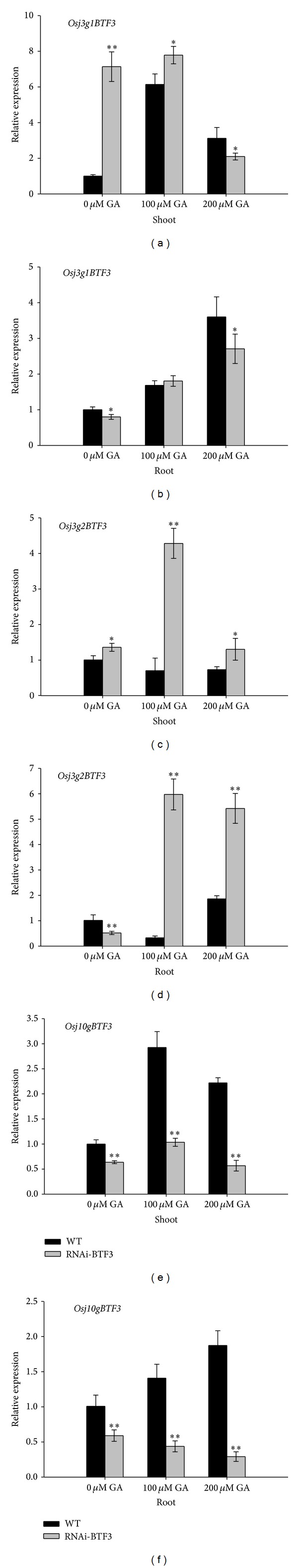
The expression analyses of* Osj10gBTF3* (LOC_Os10g34180) and its two homologous genes* Osj3g1BTF3* (LOC_Os03g01910) and* Osj3g2BTF3* (LOC_Os03g63400) in root and shoot of* Osj10gBTF3* ^Ri^ lines and wild-type (WT) under different concentrations of GA_3_. The detection was done based on three independent samples and ubiquitine was used as internal control.

The results showed that the expression of* Osj3g1BTF3* was induced by low and high concentrations of GA_3_ in root and shoot but was repressed by high concentrations of GA_3_ in shoot (Figure 5). In contrast, the number of transcripts increased with GA_3_ in* Osj10gBTF3* ^Ri^ seedling at 100 *μ*M but decreased at higher concentrations. In* Osj3g2BTF3,* samples treated with 100 *μ*M GA_3_ had less transcripts compared with controls in root. However, the transcript level significantly increased in root and shoot at 100 and 200 *μ*M GA_3_ (Figure 5). For* Osj10gBTF3* ^Ri^ lines, the transcript level increased with GA_3_ concentration both in root and in shoot but the transcript increased by about 50% in shoot and 100% in root treated with 100 *μ*M GA_3_, respectively, as compared to controls (Figure 5) and the expression of transcript increased with the increasing concentration of GA_3_ in both root and shoot (Figure 5). In contrast, the transcript level decreased in both root and shoot with GA_3_ in* Osj10gBTF3* ^Ri^ seedling particularly in root at 200 *μ*M (Figure 5).

**Figure 6:**
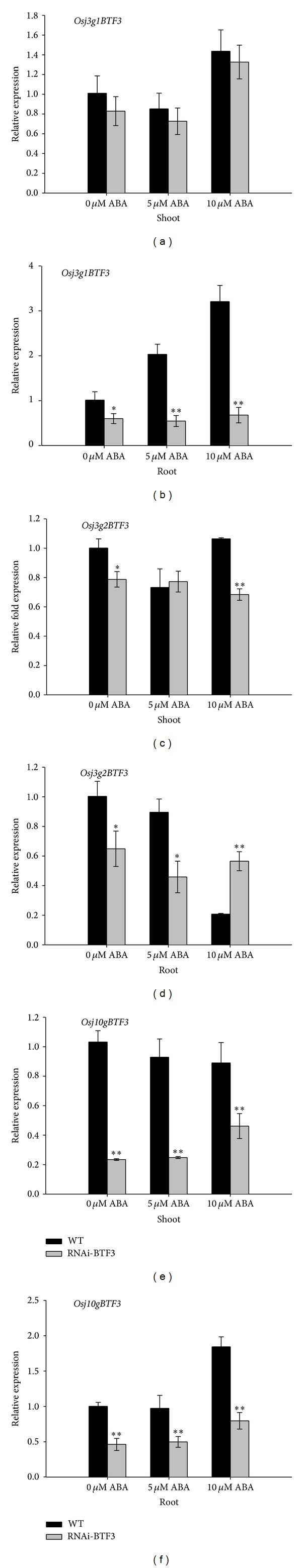
The expression analyses of* Osj10gBTF3* (LOC_Os10g34180) and its two homologous genes* Osj3g1BTF3* (LOC_Os03g01910) and* Osj3g2BTF3* (LOC_Os03g63400) in root and shoot of* Osj10gBTF3* ^Ri^ lines and wild-type (WT) under different concentrations of ABA. The detection was done based on three independent samples and ubiquitine was used as internal control.

For the ABA treatment, the number of* Osj3g1BTF3* transcripts increased with increasing level from 5 to 10 *μ*M in the root. However, the level appeared to be significantly decreased in shoot at 5 *μ*M, after which the expression level increased at 10 *μ*M (Figure 6). However, the transcript level decreased in root and shoot of* Osj10gBTF3* ^Ri^ lines after treatment with 5 *μ*M ABA, but the expression level significantly increased in root and shoot at 10 *μ*M ABA (Figure 6). In* Osj3g2BTF3,* the expression decreased with increasing level of ABA in the root and the transcript level increased significantly in shoot at 10 *μ*M ABA. In* Osj10gBTF3* ^Ri^ seedling, the expression level significantly decreased in root and shoot with increasing ABA concentration (Figure 6). Similarly, in* Osj10gBTF3*, the expression of transcript increased with increasing concentration of ABA in the root. However, the transcript level did not change significantly in shoot after treatment with ABA. In contrast, the number of transcripts decreased about 50% with ABA in* Osj10gBTF3* ^Ri^ seedling as compared to WT. These results show simply that OsBTF3 expression is mostly constitutive but slightly modulated by ABA in shoot at higher concentrations.

**Figure 7:**
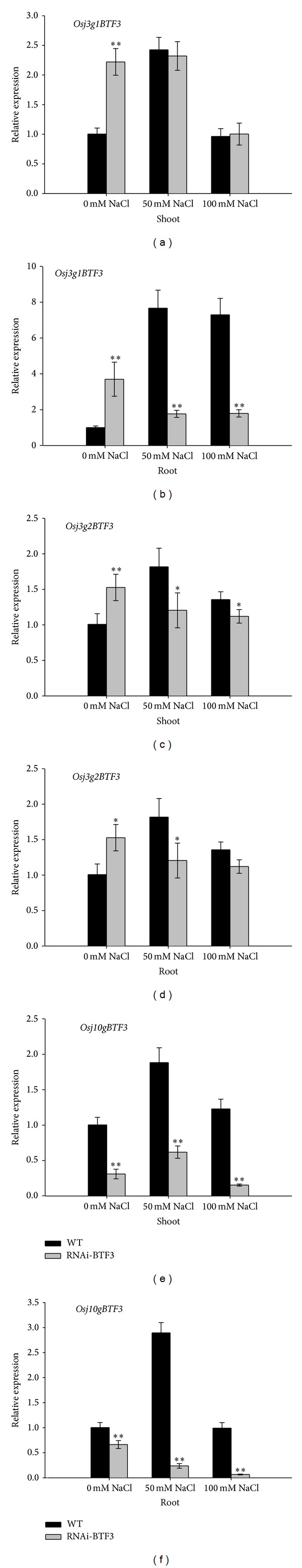
The expression analyses of* Osj10gBTF3* (LOC_Os10g34180) and its two homologous genes* Osj3g1BTF3* (LOC_Os03g01910) and* Osj3g2BTF3* (LOC_Os03g63400) in root and shoot of* Osj10gBTF3* ^Ri^ lines and wild-type (WT) under different concentrations of salt. The detection was done based on three independent samples and ubiquitine was used as internal control.

For salt treatment, the expression level of* Osj3g1BTF3* increased with increasing level of salt in the root and shoot particularly at 50 mM. In contrast, the transcript level decreased significantly in* Osj10gBTF3* ^Ri^ seedling at 100 mM NaCl (Figure 7), but the level of transcript was dramatically decreased about 80% in root at 50 and 100 mM as compared to control (Figure 7). In* Osj3g2BTF3*, the transcript level increased in both root and shoot with salt particularly at 50 mM; however, in* Osj10gBTF3* ^Ri^ lines, the level of expression gradually decreased in both root and shoot with increasing concentration as compared with WT (Figure 7). The samples treated with salt significantly upregulated the transcript level of* Osj10gBTF3* at 50 mM both in root and in shoot, after which the expression level decreased at 100 mM. In* Osj10gBTF3* ^Ri^ seedling, the number of transcripts decreased mainly in roots with salt as compared to control (Figure 7). These results show that the downregulation of the BTF3 gene in* Osj10gBTF3* ^Ri^ seedling may reflect the compromised protein translation and importing activity under salt stress. For hormone treatment, the induction effect showed by ABA was much more effective than the repression effect caused by GA_3_. There was a difference between the two hormones, indicating that BTF3 is more sensitive to GA_3_ than ABA.

## 4. Discussion

Recently, it has been documented that BTF3 plays a vital role in plant growth and development [6]. However, there are no reports available about the role of OsBTF3 and how they are regulated by GA_3_, ABA, and NaCl during seed germination and seedling growth. In the present study, BTF3-like gene* Osj10gBTF3* was cloned and functionally analyzed and the results may help to understand OsBTF3 protein function more comprehensively.

There are numerous BTF3 genes in rice genomes and in other plant species that are highly similar to each other (Figure 1(a)). The amino acid sequences of different variants of OsBTF3 are similar to other plant species excluding variations in the lengths of the exon and intron. We have observed three signal systems which include important MMTS, NLS, and ERRS in* Osj3g1BTF3* and* Osj10gBTF3*, used to target the correct destination of new synthesized proteins [6]. It has been reported that these specific addressing systems such as MMTS [16], NLS [17], and ERRS [18] have been used by the new synthesized proteins to specifically target the correct destination [19]. A NLS “RRKKK” is putatively positioned between amino acid residues 22–26 in the N-terminal (Figure 5(a)), demonstrating that the OsBTF3 protein might be located in both cytoplasmic membrane system and nucleus [6]. It is well established that BTF3 is not only a cytoplasmic membrane protein that functions in the targeting and translocation of nascent polypeptides [20, 21] but may also play an important role in the nucleus, affecting the transcription levels of some genes in rice [22–24].

It was observed that* Osj10gBTF3* and* Osj3g1BTF3* are probably similar but different from* Osj3g2BTF3* in biological function. To verify this, BTF3-like gene* Osj10gBTF3* was cloned and functionally analyzed during seed germination and seedling growth. In the present study, it was observed that the* Osj10gBTF3* ^Ri^ lines were sensitive to GA_3_, ABA, and NaCl treatment, particularly to GA_3_ since their applications did not affect final germination percentage but caused WT seeds to germinate more rapidly than the* Osj10gBTF3* ^Ri^ transgenic lines, and their mean germination rate decreased with increased concentrations of GA_3_, ABA, and NaCl. Similarly, seedling growth was inhibited compared with that of WT control, particularly when treated with 100 or 200 uM GA_3_; 50% reduction in shoots were observed in* Osj10gBTF3* ^Ri^ seedlings (Figure 5). RNAi inhibition of* Osj10gBTF3* reduced the expression of* Osj10gBTF3* transcripts, as a result reducing the manufacturing of the essential amount of NAC complex for normal plant growth. Hence, once the transcript level of* Osj10gBTF3* is decreased, it disturbed the function of this monomeric subunit plus the complete NAC, resulting in severe vegetative defects in* Osj10gBTF3* ^Ri^ lines [6]. It has been reported that the repression of* Osj10gBTF3* caused serious plant growth and development defects [6]. These results are similar to some other studies demonstrating that BTF3 take part in cell cycle regulation via interaction with cyclins [25]. Mutants lacking NAC also showed visible growth and morphological problem in yeast and tobacco [20, 26]. Furthermore, the inhibition of BTF3 participated in the repression of transcription and protein synthesis in apoptotic K562 cells [27], while change in BTF3 expression is linked with apoptosis in BL60 Burkitt lymphoma cells [28]. On the basis of these results, we can conclude that BTF3 plays an important role in seed germination and seedling growth.

To further verify these results, we observed expression of different variants of OsBTF3 in root and shoot in* Osj10gBTF3* ^Ri^ and WT seedlings. We observed that the expression of different variants of OsBTF3 was primarily constitutive, being generally modulated by different GA_3_, ABA, and salt stress treatments in* Osj10gBTF3* ^Ri^ and WT seedlings (Figures 5, 6, and  7). The expression of* Osj3g1BTF3* and* Osj10gBTF3* was induced by low and high concentrations of GA_3_ and ABA in root and shoot (Figures 5 and 6), but the transcript level decreased in shoot at high concentrations (Figures 5 and 6). In* Osj10gBTF3* ^Ri^ seedling, the transcript level increased both in root and in shoot with increasing GA_3_ and ABA concentration, whereas the transcript level of* Osj10gBTF3* decreased with increasing GA_3_ concentration (Figures 5 and 6). In* Osj3g2BTF3,* seedling treated with 100 *μ*M GA_3_ had less transcripts, but the level significantly increases in root at 100 *μ*M GA_3_ (Figure 5), whereas, in* Osj10gBTF3* ^Ri^ lines, the transcript level increased both in root and shoot with GA_3_ concentration (Figure 5). In* Osj3g2BTF3* the transcript levels decreased with increasing level of ABA in the root but increased in shoot at 10 *μ*M ABA. In* Osj10gBTF3* ^Ri^ seedling, the expression level significantly decreased in root and shoot with increasing ABA concentration (Figure 6). Wang et al. [6] observed a 62% increase in* Osj10gBTF3* expression level with ABA, compared to a 22% reduction with GA_3_ after treatment with the same concentration for 12 h. In previous reports, it has been observed that the transcript level of* NbBTF3* remained unaffected when exposed to 100 *μ*M of ABA and GA_3_ [26]. Similarly, He et al. [29] did not find any significant change in* SmBTF3,* when treated with ABA for 24 hrs. By using 100 *μ*M of ABA to treat maize (*Zea mays* L.) plants, Zhang et al. [30] found that* ZmBTF3* (*Zea mays BTF3*) expressions were significantly decreased. The salt concentration upregulated* Osj3g1BTF3, Osj3g2BTF3,* and* Osj10gBTF3* expression at 50 mM in both root and shoot, but, in* Osj10gBTF3* ^Ri^ seedling, the number of transcripts decreased mainly in roots with salt as compared to control (Figure 7). Wang et al. [6] have observed that the expression of* Osj10gBTF3* was downregulated under high salt concentration, while low salt concentrations upregulated* Osj10gBTF3* expression in rice. Similarly, Li et al. [4] demonstrated that transcript level of OsBTF3 was significantly reduced in rice plants under salt stress and the salt resistance was improved in the seedlings of OsBTF3 transgenic lines. In maize, Zhang et al. [30] observed that expression of BTF3 was downregulated at 250 mM NaCl. In contrast, BTF3 transcript level was significantly increased by salt stress in* Suaeda asparagoides* [31], while expression level of* SabBTF3* was differentially regulated by various abiotic stresses, such as drought, salinity, ABA, and temperature in roots [7]. These results also suggested that* Osj3g1BTF3* and* Osj10gBTF3* are much similar in their response to GA_3_, ABA, and NaCl during seedling stage.

This is a first report demonstrating the involvement of different variants of BTF3 in seed germination and seedling stage. It was observed that* Osj3g1BTF3* and* Osj10gBTF3* are a similar type of genes which plays an important role in plant growth, especially in seed germination and seedling growth. Functional analysis revealed that both of these genes are much similar in biological functions. Further investigation is required to provide the insight into the functions of BTF3 and the connected mechanisms responsible for improving abiotic stress tolerance in plants.

## Supplementary Material

Detail of the proteins sequences used for phylogenetic analysis in this study are given in the Supplementary Table 1. While the detail of forward and reverse primers which were used are given in Supplementary Table 2.
