# Supplementary material for: Basal Transcription Factor 3 Plays an Important Role in Seed Germination and Seedling Growth of Rice
Source: Biomed Res Int. 2014 May 29;2014:465739. doi: 10.1155/2014/465739 (PMC4058115; doi:10.1155/2014/465739)
Supplement: Supplementary file 1 — Detail of the proteins sequences used for phylogenetic analysis in this study are given in the Supplementary Table 1. While the detail of forward and reverse primers which were used are given in Supplementary Table 2. [file 465739.f1.pdf]

Supplementary Table 1: List of proteins used for phylogenetic analysis in this study.

Species	Protein name	Accession No. in NCBI
Oryza sativa	Osj3g1BTF3	NP_001048709
	Osj3g2BTF3	NP_001051911
	Osj10gBTF3	NP_001064883
Sorghum bicolor	SbBTF3	EER93008
Arabidopsis thaliana	AtBTF3	AEE29647
Nicotiana benthamiana	NbBTF3	ABE01085
Capsicum annuum	*CaBTF3*	ABM55742
Ricinus communis	*RcBTF3*	EEF34688
Solanum lycopersicum	*SlBTF3*	NP_001234229
Triticum aestivum	*TaBTF3*	AFV31408
Zea mays	*ZmBTF3*	ACG28870

Supplementary Table 2: List of Q-PCR primers, which were used in the experiment.

Gene name (Accession No.)	Primer	Sequence (5′→3′)
Osj10gBTF3 (LOC_Os10g34180)	BTF3-10-F	CTCCTGGTGCTTCTGGT
	BTF3-10-R	GGCAATTCGCAACTTAT
Osj3g1BTF3 (LOC_Os03g01910)	BTF3-3-1-F	TTGCTGAGCAGTTCCAGA
	BTF3-3-1-R	CTAGGACGACTCTTTCTTCTC
Osj3g2BTF3 (LOC_Os03g63400)	BTF3-3-2-F	TCCTGGGATCATTAACCAAT
	BTF3-3-2-R	TCAGGCTTTTGTCTCCTGAG
	UBI-F	CTGTCAACTGCCGCAAGAAG
	UBI-R	GGCGAGTGACGCTCTAGTTC
